# New Anti-Nodal Monoclonal Antibodies Targeting the Nodal Pre-Helix Loop Involved in Cripto-1 Binding

**DOI:** 10.3390/ijms160921342

**Published:** 2015-09-07

**Authors:** Annalia Focà, Luca Sanguigno, Giuseppina Focà, Luigi Strizzi, Roberta Iannitti, Rosanna Palumbo, Mary J. C. Hendrix, Antonio Leonardi, Menotti Ruvo, Annamaria Sandomenico

**Affiliations:** 1 Institute of Biostructure and Bioimaging, National Research Council (IBB-CNR) and Centro Interuniversitario di Ricerca sui Peptidi Bioattivi (CIRPeB), Università degli Studi di Napoli “Federico II”, Naples 80134, Italy; E-Mails: annalia.foca@gmail.com (A.F.); giuseppina.foca@gmail.com (G.F.); robertaiannitti@gmail.com (R.I.); rosanna.palumbo@cnr.it (R.P.); 2 Department of Pharmacy, Università degli Studi di Napoli “Federico II”, Naples 80131, Italy; 3 Bioker Multimedica, Naples 80131, Italy; E-Mail: Luca.Sanguigno@multimedica.it; 4 Program in Cancer Biology and Epigenomics, Stanley Manne Children’s Research Institute at Ann and Robert H. Lurie Children’s Hospital of Chicago, Robert H. Lurie Comprehensive Cancer Center, Northwestern University Feinberg School of Medicine, Chicago, IL 60611, USA; E-Mails: LStrizzi@luriechildrens.org (L.S.); m-hendrix@northwestern.edu (M.J.C.H.); 5 Department of Molecular Medicine and Medical Biotechnologies, Università degli Studi di Napoli “Federico II”, Naples 80131, Italy

**Keywords:** Fab fragments, monoclonal antibody, melanoma, nodal, SPR

## Abstract

Nodal is a potent embryonic morphogen belonging to the TGF-β superfamily. Typically, it also binds to the ALK4/ActRIIB receptor complex in the presence of the co-receptor Cripto-1. Nodal expression is physiologically restricted to embryonic tissues and human embryonic stem cells, is absent in normal cells but re-emerges in several human cancers, including melanoma, breast, and colon cancer. Our aim was to obtain mAbs able to recognize Nodal on a major CBR (Cripto-Binding-Region) site and to block the Cripto-1-mediated signalling. To achieve this, antibodies were raised against *h*Nodal(44–67) and mAbs generated by the hybridoma technology. We have selected one mAb, named 3D1, which strongly associates with full-length *rh*Nodal (*K*_D_ 1.4 nM) and recognizes the endogenous protein in a panel of human melanoma cell lines by western blot and FACS analyses. 3D1 inhibits the Nodal-Cripto-1 binding and blocks Smad2/3 phosphorylation. Data suggest that inhibition of the Nodal-Cripto-1 axis is a valid therapeutic approach against melanoma and 3D1 is a promising and interesting agent for blocking Nodal-Cripto mediated tumor development. These findings increase the interest for Nodal as both a diagnostic and prognostic marker and as a potential new target for therapeutic intervention.

## 1. Introduction

Nodal ligands are potent morphogens, belonging to the TGF-β superfamily [1,2,3,4]. In humans, Nodal is rarely expressed during late development and adulthood. Nodal physiological expression is typically observed only in embryonic tissues and embryonic stem (ES) cells. Aberrant re-activation of Nodal expression in adults is associated with a number of tumors such as metastatic melanoma as well as breast, colon, prostate, and ovarian carcinomas [5,6,7,8].

During the last years, increased attention has been devoted to the correlation existing between the expression levels of Nodal and melanoma development. These studies have associated the hyper-activation of Nodal signalling to melanoma invasiveness, aggressiveness, plasticity, and tumorigenicity [5,9,10,11,12,13].

In human metastatic melanoma cells, the up-regulation of Nodal is controlled by the epigenetic silencing of the antagonist Lefty [10,14,15] and by a positive cross-talk with the Notch4 ligand [16,17].

Under physiological conditions, Nodal signalling plays a crucial role in early embryonic and neural development and in the maintaining of pluripotency in ES cells. It is synthesized by cells as pre-pro-Nodal (55 kDa) and subsequently converted to the intermediate pro-Nodal (37 kDa) and finally to the mature and active Nodal (22 kDa) by a proteolytical process induced by subtilisin-like proprotein convertases PACE-4 and Furin. Once secreted from cells, Nodal can act as either autocrine or paracrine agent.

Nodal signalling is triggered by interaction with heteromultimeric complexes consisting of the EGF-like domain of the co-receptor Cripto-1 and type I (ALK 4/7) and type II (ActRIIB) activin-like serine-threonine kinase receptors. Such association leads to the phosphorylation and activation of ALK 4/7 by ActRIIB which, in turn, promotes the phosphorylation of Smad2/3. Hence, phosphorylated Smad-2/3 proteins associate with the common mediator Smad-4 prior to translocation to the nucleus where, together with transcription factors such as FoxH1, Mixer, and p53, regulate expression of some target genes. These latter include Nodal as well as Lefty A and B, which are natural extracellular inhibitors. Interestingly, Nodal activates its own expression via a positive feedback loop.

Nodal signalling can occur in the extracellular microenvironment, where Nodal and Cripto-1 are present as free ligands, as well as on the cellular membrane surface, in the form of a multimeric complex. It is well established that Cripto-1 is essential for the cellular activation of Nodal/ALK4/Smad pathway [18] and that it may directly interact with ALK4 to enhance the responsiveness of the ALK7/ActRIIB complex to Nodal [19]. Recently we have reported that Nodal is also capable of physically interacting with both ALK7 and ALK4 regardless of the Cripto-1 presence [20,21]. Although the exact mechanisms underlying the synergistic effect exerted by Cripto-1 on the cellular activation of Nodal-dependent Smad signalling have not been completely understood, the detection of Cripto-1 in melanoma cells [5,22,23] supports the relevant role this co-receptor can play in co-promoting tumorigenesis.

Like other ligands of the TGF-β superfamily, Nodal can activate additional signalling pathways such as MAPK/ERK (ras/raf/mitogen-activated protein kinase) and PI3K–AKT. MAPK signalling, which converges on ERK and is related to the single mutation in the BRAF protein, has been also reported as a relevant pathway involved in melanoma disease and progression [24,25].

These findings suggest that the quantification of Nodal expression in cancer tissues is both a diagnostic and prognostic option and designate the protein as a biomarker and as a potential new therapeutic target in melanoma and other tumors.

Multiple pharmacological therapies have been approved for malignant melanoma, including BRAF inhibitors (vemurafenib and dabrafenib), MEK inhibitors (trametinib), mAbs, such as Ipilimumab (mAb anti-CTLA4) and Pembrolizumab (mAb anti-PD1), and the oldest chemotherapy drug (dacarbazine). However, many of these show a limited efficacy due to the onset of drug resistance mechanisms or their low specificity [25,26,27,28,29,30]. The identification of more effective anti-tumor agents against novel targets, to be employed alone or in combination with those already available, remains an interesting and challenging approach. In this context, targeting and inhibition of Nodal signalling represents an attractive and alternative strategy to block melanoma progression and potentially of other cancers. With this aim, we have generated monoclonal antibodies against the Nodal 44–67 region which has been recommended as a major site of Cripto-1 recognition and as one of the key structural elements of the Nodal-Cripto-ALK4 interactions [21,31]. Using a subtractive ELISA screening we have selected a monoclonal antibody that recognizing the Nodal E49 and E50 hot-spot residues, and neutralizes *in vitro* the binding to Cripto-1 in a specific way. We report here a full biochemical characterization of the antibody and of its functional fragments, together with preliminary data showing the selective detection of the endogenous protein in a set of human melanoma cells. Given the potential to selectively inhibit the Nodal-Cripto-Smads axis, this antibody could represent a distinctive option to block cancer progression in Nodal-positive tumor tissues [32].

## 2. Results

### 2.1. Antigen Design

**Figure 1:**
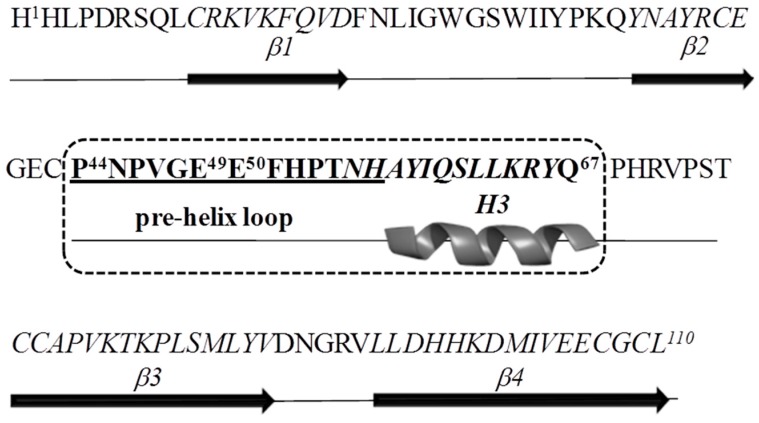
Amino acid sequence and secondary structure of the Nodal monomer. The Nodal antigen used to generate the monoclonal antibodies is in bold and boxed by a dashed line. The epitope recognized by 3D1 is underlined. Residues E49 and E50 involved in the binding to Cripto-1 are numbered.

On the basis of previous docking and binding studies [21,31] the region of human Nodal (Uniprot Q96S42) including the H3-wrist helix and the pre-helix loop was chosen as *h*Nodal antigen (Figure 1). As reported, the *h*Nodal(44–67) peptide, referred to the 1–110 residues numbering and corresponding to the mature form of the endogenous protein (238–347 a.a.), is involved in the binding to the co-receptor Cripto-1 and contains two glutamic acid residues, E49 and E50, that crucially stabilize the interaction. With the aim to select anti-Nodal antibodies able to recognize these two hot-spot residues, we devised a simple and novel strategy of screening. After immunization and hybridoma generation, cell supernatants were screened using in parallel a mutated peptide named *h*Nodal(44–67)E49A–E50A, in which E49 and E50 were replaced with two alanines.

### 2.2. Chemical Synthesis, Purification, and Identification of Human Nodal and GDF Peptides

*h*Nodal(44–67) and the mutated variants were synthesized with either a free amino group at the N-terminus (used for conjugation with KLH and immunogen preparation), or acetylated and amidated to better mimic the peptide within the protein structure.

A set of mutated variants of *h*Nodal(44–67), reported in Table 1, was designed to define the epitope and to investigate the contribution of specific residues to the recognition with antibodies. Peptides from 1 to 6 (Table 1) included variants of *h*Nodal(44–67) bearing doubly mutated residues at the N-terminus; shorter peptides (entries from 4 to 6, Table 1) were used to confirm the epitope on the N-terminal region. Peptides reported in entries 7–9 of Table 1, Nodal(1–38), Nodal(39–75), and Nodal(76–110), were prepared as reported in *Saporito et al.* [33] and used to confirm the specificity of antibodies for the Nodal internal fragment.

Peptides 10 and 11 in Table 1, reproducing the region 433–445 of human GDF5 (Uniprot, P43026), region 386–398 of human GDF 6 (Uniprot, Q6KF10) and region 382–394 of human GDF 7 (Uniprot, Q7Z4P5), belonging to Growth Differentiation Factors (GDF family), which are close structural homologs of Nodal [34], were tested to explore the selectivity of antibodies among TGF-β family ligands. Sequences were chosen on the basis of multiple alignment of *h*Nodal(44–67) versus all TGF-β ligands performed by using the BLAST server.

All peptides were synthesized, purified, and identified as described in the Methods section; they were obtained with an average yield of 50% and with a purity level above 95%, as determined by LC–MS analysis (Table 1).

**Table 1:** Nomenclature, amino acid sequence, and *M*w (calculated and experimental) of *h*Nodal and *h*GDF peptides.

#	*h*Nodal peptide	Sequence	*M*w Theor.* (amu)	*M*w Exp.** (amu)
**1**	(44–67)	PNPVGEEFHPTNHAYIQSLLKRYQ	2878.44	2879.3
**2**	(44–67) E49A-E50A	PNPVGAAFHPTNHAYIQSLLKRYQ	2762.43	2763.5
**3**	(44–67) P46A-V47A	PNAAGEEFHPTNHAYIQSLLKRYQ	2824.39	2825.5
**4**	(44–56)	PNPVGEEFHPTNH	1617.68	1617.8
**5**	(52–60)	HPTNHAYIQ	1120.53	1121.2
**6**	(56–67)	AYIQSLLKRYQ	1422.78	1423.5
**7**	(1–38)	HHLPDRSQLCRKVKFQVDFNLIGWGSWIIYPKQYNAYR	4716.43	4718.4
**8**	(39–75)	CEGECPNPVGEEFHPTNHAYIQSLLKRYQPHRVPSTC	4276.98	4278.7
**9**	(76–100)	CAPVKTKPLSMLYVDNGRVLLDHHKDMIVEECGCL	3966.95	3968.6
**10**	*h*GDF 5 ***	CEFPLRSHLEPTNH	1719.80	1719.8
**11**	*h*GDF 6/7	CDFPLRSHLEPTNH	1705.78	1705.7

# Peptide number; * Theor.: Theoretical; ** Exp.: Experimental; *** GDF: Growth Differentiation Factor.

### 2.3. Screening of Hybridoma Supernatants and Epitope Identification

**Figure 2:**
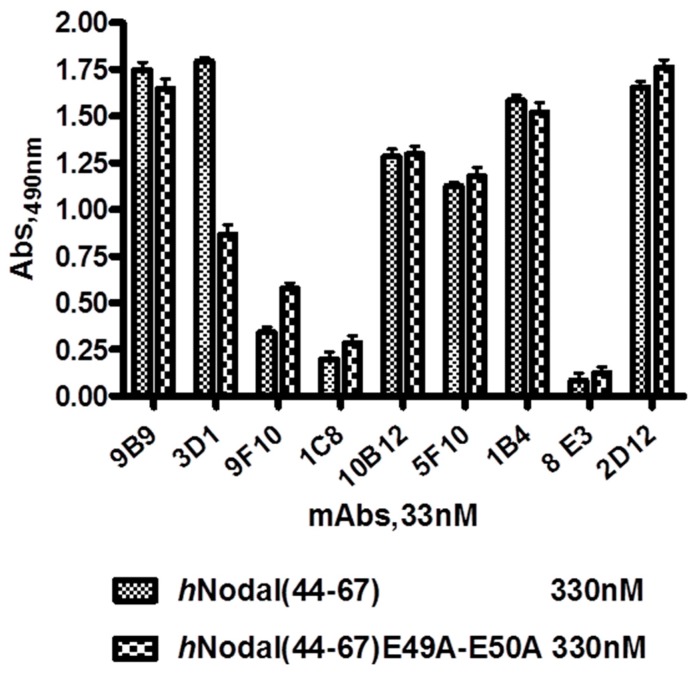
ELISA-based screening assay of hybridoma supernatants. *h*Nodal(44–67) and *h*Nodal(44–67)E49A–E50A were coated at 1.0 µg/mL (330 nM). Hybridoma supernatants were tested at 5.0 µg/mL total protein (33 nM assuming the occurrence of purified antibodies).

An initial screening of hybridoma supernatants was carried out by ELISA coating the *h*Nodal(44–67) BSA-conjugated peptide. By this preliminary test, nine clones were positive. Following our strategy, the pre-selected hybridoma supernatants were screened using in parallel *h*Nodal(44–67) and its mutated variant *h*Nodal(44–67)E49A–E50A. The assay was performed coating the unconjugated peptides at 330 nM and testing the hybridoma supernatants at a total protein concentration of 5.0 µg/mL (33 nM). Data reported in Figure 2 show that supernatants from six clones, indicated as 9B9, 3D1, 10B12, 5F10, 1B4, and 2D12, although to a different extent, recognized the immobilized antigen. Clones 9F10, 1C8, and 8E3 showed a very weak signal and were therefore not further considered. The clone named 3D1 recognized the wild-type *h*Nodal(44–67) peptide much better than the mutated variant, suggesting that binding occurs close to the region encompassing the two glutamic residues crucial for the binding of Nodal to the co-receptor Cripto-1. 3D1 clone was thereby identified as the most promising to show a neutralization activity for the interaction Nodal/Cripto-1.

### 2.4. SPR Binding Analyses of mAbs to rhNodal Protein

After selection, the ability of the purified monoclonal antibodies to bind the full-length recombinant human Nodal was evaluated and considered as a pre-requisite for their use as diagnostic tools. The screening was carried out by SPR direct binding. MAbs were initially tested at 100 nM; at this concentration only two antibodies, 3D1 and 5F10, were able to bind the full-length *rh*Nodal protein (Figure S1). To estimate their affinity constant, dose-dependent binding experiments were next carried out. Analyses were performed injecting increasing doses of antibodies determining for each run association and dissociation rate constants. 3D1 bound Nodal with a *K_D_* value of 1.42 nM, whereas 5F10 was characterized by a weaker affinity (83 nM, see Table S1). The 3D1 displayed rapid association (average *k_a_* = 6.95 × 10^5^ M^−1^·s^−1^) and slow dissociation rates constants (average *k_d_* = 6.55 × 10^−4^ s^−1^), resulting in a high binding affinity to the protein. 5F10 exhibited a lower affinity as result of a slower association (average *k_a_* = 1.91 × 10^4^ M^−1^·s^−1^) and quicker dissociation rate (average *k_d_* = 1.08 × 10^−3^ s^−1^). Binding curves for the two mAbs are reported in Figure S1b,c. Kinetics parameters are reported in Table S2a,b.

### 2.5. Production and Purification of 3D1 F(ab′)_2_/Fab′ Fragments

In the attempt to produce smaller antibody fragments useful for crystallization studies or as additional reagents for Nodal detection, we tried to obtain 3D1-derived Fab fragments by enzymatic digestion. 3D1 was first deglycosylated with PNGase F to remove a single *N*-linked glycan at Asn^297^ on each CH2 domain of the two heavy chains [35]. After deglycosylation, the whole antibody was digested with pepsin. Digestion was completed after four hours of incubation. As shown (Figure S2), pepsin converted 3D1 into F(ab′)_2_, with a MW of about 110 kDa, without formation of other fragments. F(ab′)_2_ fragment was next isolated from the digestion mixture by a two-step purification procedure that included Protein G affinity and size-exclusion chromatography (See Figure S3a). The SEC chromatographic profile showed absence of aggregates and the presence of a highly homogenous product; moreover, its elution volume was in agreement with the expected molecular weight (~110 kDa, See Figure S3c). Integrity and purity of the products were confirmed after each step by SDS-PAGE analysis under reducing and non-reducing conditions, Figure S3b,d respectively. Next, Fab′ fragment was obtained by selective reduction of the hinge-region disulfide bonds of F(ab′)_2_ using mercaptoethylamine. Reduction was successfully achieved after three hours, as shown by SDS-PAGE analysis under non-reducing conditions (Figure S4a). To carry out further analyses, the Fab′ was alkylated with IAM, then it was purified by size-exclusion chromatography to confirm its identity and to evaluate the presence of potential aggregates. As indicated by the single peak eluted at 13.65 mL (Figure S4b), no aggregates were detected. 3D1 Fab′ was also characterized by LC–ESI-TOF MS after selective reduction of the disulfide bridge connecting the light and the heavy chain. LC–MS analysis of the separated chains reported in Figure S5a–c show a single mass peak for the light chain and four prevailing peaks for the heavy chain accounting for incomplete IAM derivatization (mass difference of 57 Da) and a double splitting at level of the hinge region. On the basis of the mass difference of 186 Da observed between the two main peaks and the highly conserved sequences of mouse IgG1 within the hinge region [36,37], see also Figure S5d, we have hypothesized that pepsin operates two cleavages just before and after the S^116^V^117^ residues (UniProtKB, P01868), as indicated by the arrows.

### 2.6. SPR Comparative Binding Analyses of 3D1 F(ab′)_2_/Fab′ Fragments

**Figure 3:**
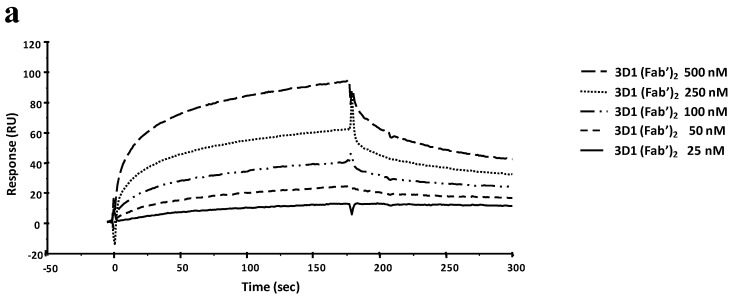

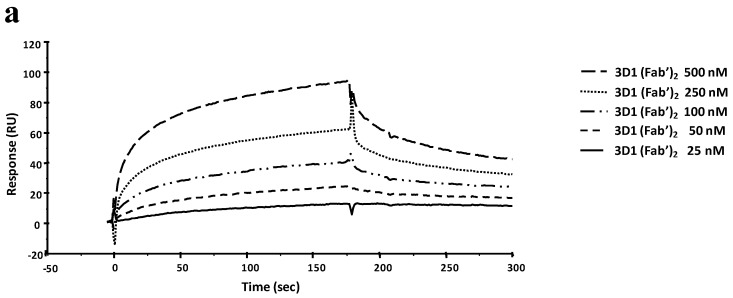
Overlay plot of SPR sensorgrams showing the binding of the 3D1 F(ab′)_2_ (**a**) and Fab′ (**b**) to *rh*Nodal immobilized on a CM5 sensor chip. The interaction was monitored at concentrations of F(ab′)_2_ ranging between 25 and 500 nM, and of Fab′ ranging between 25 and 200 nM, obtaining dose-dependent binding curves.

The ability of the F(ab′)_2_ and Fab′ fragments to bind to *rh*Nodal protein was assessed by a comparative binding assay carried out by SPR. The 3D1 F(ab′)_2_ and Fab′ fragments bound the immobilized *rh*Nodal with similar association and dissociation rate constants, thus also the same affinity (*K_D_* = 15 nM, Figure 3a,b). This *K_D_* value is 10-fold higher compared to that exhibited by the whole antibody (*K_D_* = 1.4 nM), thereby the affinity is 10-fold lower. Kinetic parameters are reported in Table S2c–d.

### 2.7. Epitope Mapping Study

**Figure 4:**
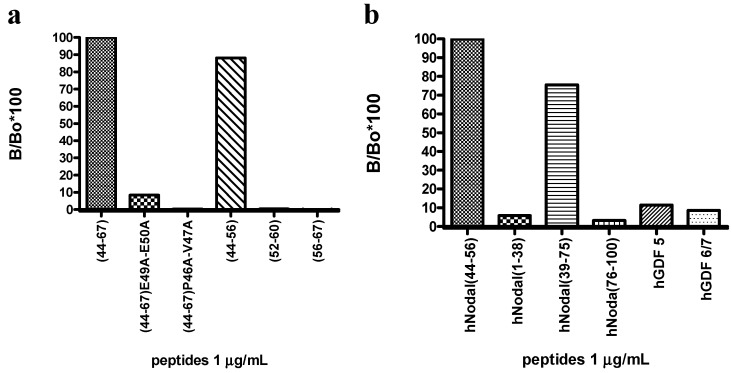
Characterization of the 3D1 binding properties. (**a**) Mapping of 3D1 mAb epitope; peptides were coated at 1.0 µg/mL; (**b**) Bar graph showing the specificity of the 3D1 for the central region of human Nodal; peptides were coated at 1.0 µg/mL. Absorbance value of each peptide (B) was normalized to the (44–67) peptide, assumed as 100% of signal (B_0_). The signal was expressed as % of relative absorbance measured at 490 nm and calculated as B/B_0_ × 100.

To finely delineate the epitope recognize by the 3D1 antibody, a mapping of the original antigen was carried out by ELISA and SPR analyses. For this purpose, the peptide antigen and its mutated variants (see Table 1) were tested by ELISA and SPR for binding to 3D1 and to functional fragments. ELISA assays carried out by coating the different peptides at the same concentration showed that the strongest signal was detected with the short (44–56) peptide (Figure 4a), whereas weaker signals were obtained with variants bearing the mutated E49 and E50. In addition, no binding was observed with variants in which P46 and V47 were mutated to alanines, as well as with other N-terminally truncated shorter peptides.

These data confirmed the high specificity of 3D1 for the N-terminal residues, specifically P46, V47, E49, and E50. Subsequently, SPR dose-response binding assays were performed with peptides (44–67) and (44–56) to extrapolate *K_D_* values (See Figure S6a,b and Figure S7a,b). In Table S3 relevant data obtained by these analyses are reported. They confirm that region (44–56) contains the epitope recognize by 3D1 mAb and that residues from 46 to 50 are the most crucial for binding. Notably, the region falls within the pre-helix loop, encompassing the two glutamic acid residues crucial for the binding of Nodal to Cripto-1. The data suggest that 3D1 does not recognize a conformational epitope but rather a linear epitope.

### 2.8. Specificity Assay

ELISA assays were performed to further assess the specificity of the 3D1 mAb for the region of Nodal(44–56) involved in the binding with the co-receptor Cripto-1. New Nodal peptides were therefore screened for binding to 3D1. These peptides were: *h*Nodal(1–38), mimicking the protein N-terminal portion; *h*Nodal(39–75), mimicking the central region and *h*Nodal(76–110) mimicking the C-terminal portion of the mature form of human Nodal. Other peptides mimicking the region of the TGF-β ligand GDF and matching the 44–67 region of Nodal (GDF5 and GDF6/7, see Table 1) were also used for this purpose. As shown in Figure 4b, only fragment (39–75) of Nodal, containing the region 44–67 used to generate the antibody, did bind with similar efficiency 3D1, whereas the other peptides were essentially unreactive.

To further confirm the specificity of 3D1 binding to its antigen and to the full length protein, we used *h*Nodal(44–56) to block the staining of endogenous Nodal expressed in human embryonic stem cells. The experiment was performed by probing the blots with either 3D1 alone and after pre-incubation with the peptide. As reported in Figure S8, both forms of endogenous Nodal were no longer detectable when the mAb was pre-incubated with the peptide, suggesting that 3D1 recognizes the same epitope also on the native protein. This confirmed the selectivity of 3D1 for the Nodal epitope.

### 2.9. Detection of Endogenous Nodal Protein

**Figure 5:**
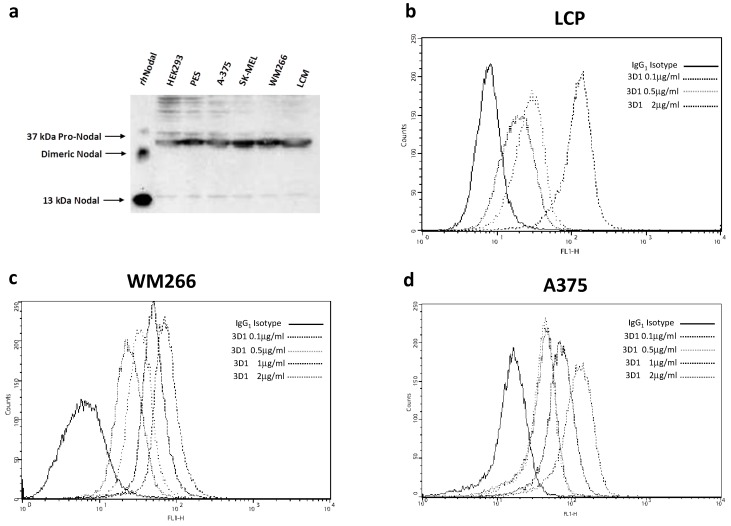
(**a**) Western blot analysis of melanoma cell lysates resolved by 15% SDS-PAGE under reducing conditions. As positive control HEK-293 cells and 100 ng of *rh*Nodal were loaded. 3D1 antibody was used at 2.0 µg/mL. Detection was achieved using GAM-HRP antibody and ECL as substrate. Cytofluorimetric staining of Nodal in human melanoma cell lines LCP (**b**), A375 (**c**) and WM266 (**d**) using 3D1. Data were collected after cell fixation and permeabilization. An unrelated IgG_1_ isotype antibody was used as negative control.

To evaluate the use of the 3D1 as a diagnostic reagent, western blotting analyses were carried out to explore the ability of the mAb to recognize the endogenous forms of Nodal protein in human melanoma cells. As shown in Figure 5a, 3D1 was able to stain the immature form pro-Nodal (approximately 37 kDa) in a panel of human melanoma cell lines and in non-melanoma HEK-293 cells, used as positive control. The smaller mature form of Nodal, approximately 13 kDa, was only barely detected, suggesting it is likely highly unstable or that this form is mostly secreted. The capability of 3D1 to recognize endogenous Nodal in melanoma cell lines was also confirmed by cytometric analyses. FACS analyses (Figure 5b–d) showed that 3D1 bound native Nodal protein also in intact cells at very low concentrations (0.1 µg/mL) and in a dose-dependent manner, as demonstrated by the increase of signal intensity with increasing antibody concentration. We could not establish by these data which form of Nodal was detected in melanoma cancer cell lines, since both mature and pro-Nodal are detected by 3D1 in western blot analyses (Figure 5a).

## 3. Experimental Section

### 3.1. Peptide Synthesis, Purification, and Identification

The wild-type *h*Nodal(44–67) peptide, its mutated variants and human GDF peptides 5, and 6/7, were prepared by step-wise solid phase synthesis as C-terminally amidated variants following standard Fmoc chemistry using an automatic SYRO system (MultiSynTech, Witten, Germany). A Rink-amide MBHA resin (Novabiochem, Laufelfingen, Switzerland) with a substitution grade of 0.57 mmol/g and amino acid derivatives with standard protections were used in all syntheses. Polypeptides were assembled under canonical conditions of peptide synthesis, using for each coupling reaction HATU/DIEA pre-activation and a five-fold excess of Fmoc-protected amino acids. Coupling and deprotection times were kept at 30 and 20 min, respectively. Standard side-chain protection groups for Fmoc chemistry were used for all residues [38]. N-terminal acetylation was performed on the resin using acetic anhydride at 0.5 M in DMF with 5% DIEA, 20 min at room temperature. The cleavage of peptides from the solid support was performed by treatment with a trifluoroacetic acid (TFA)/tri-isopropylsilane (TIS)/water (90/5/5, *v*/*v*/*v*) mixture for 90 min at room temperature. Crude peptides were precipitated in cold di-ethyl-ether, dissolved in a water/acetonitrile (1/1, *v*/*v*) mixture and lyophilized. After lyophilisation, peptides were dissolved in a solution of H_2_O/CH_3_CN 95/5 *v*/*v*, containing 0.1% TFA. They were then purified by reverse-phase HPLC (RP-HPLC) on a WATERS Prep 150 LC preparative system (Waters, Milan, Italy) using a semi-preparative 10 × 1 cm ID C18 monolythic Onyx column (Phenomenex, Torrance, CA, USA), applying a linear gradient of 0.05% TFA in CH_3_CN from 5% to 70% over 10 min at a flow rate of 15 mL/min, and monitoring the absorbance at 214 nm. The collected fractions were lyophilized. Peptide purity and identity were confirmed by liquid chromatography–mass spectrometry analysis (LC–MS); analyses were carried out on a LCQ DECA XP ion Trap mass spectrometer (ThermoFisher, Waltham, MA, USA) equipped with an OPTON ESI source and with a complete Surveyor HPLC system. Typical gradients applied to elute the peptides were as follows: flow rate 0.2 mL/min; gradients from 5% solvent B (ACN, 0.05% TFA) to 60% solvent B in 10 min. Solvent A was H_2_O, 0.08% TFA. Biobasic C18 50 × 2 mm ID columns (ThermoFisher, Milano, Italy) were used to separate peptides during LC–MS analyses.

### 3.2. Immunogen Preparation

One mg of non-acetylated *h*Nodal(44–67) peptide was conjugated with 3.0 mg carrier proteins (KLH and BSA) in 2.0 mL of 20 mM phosphate buffer pH 7.0 containing 0.2% *v*/*v* glutaraldehyde (stock solution 25%), by stirring the mixture for 3 h at room temperature [39]. The reaction was blocked by adding 1.0 mL of 1.0 M glycine in water, then solutions were extensively dialyzed against PBS buffer pH 7.4 before being lyophilized. The amount of peptide-protein conjugate was determined using the Bradford assay [40].

### 3.3. Antibody Generation

BALB/c mice were housed and handled according to the institutional guidelines (Project identification code 2013/0038120, approved by the Ethical Animal Care and Use Committee, University of Naples “Federico II”. Date of approval 24 April 2013). Four five-week old female BALB/c mice (Jackson Lab) were immunized by sub cutaneous injection with 300 μL of suspension containing 100 μg of KLH-conjugated *h*Nodal(44–67) peptide mixture emulsified with Complete Freund’s Adjuvant. Before immunization, 250 µL blood samples were taken from each mouse from the caudal vein and used as the pre-immune control (T0 samples). Mice were boosted with the same amount of immunogen in incomplete Freund’s adjuvant at day 18 and at day 30; blood samples were taken from the caudal vein (250 µL) before every subsequent immunization and tested by ELISA to monitor antibody titer. A final antigen boost was administered sub-cutis in to the mice showing the highest antibody titer seven days before being sacrificed and splenectomised as described below. Cells harvested from spleens of sacrificed animals were fused with myeloma SP2/0 (ATCC) cells at a ratio of 5:1 in RPMI-GM containing polyethylene glycol (PEG) 1300–1600 (Hybri-Max, Sigma–Aldrich, Milano, Italy) and 7.5% DMSO (Sigma–Aldrich, Milano, Italy) as described by Köhler [41].

The fused hybridoma cells were re-suspended in 30 mL of selection medium consisting of RPMI–GM medium containing 10% FBS, 100 U/mL penicillin, 100 μg/mL streptomycin, 100 μM hypoxanthine, 16 μM thymidine and 400 nM aminopterin (RPMI-HAT Sigma–Aldrich, Milano, Italy). The cell suspension (200 μL) was dispensed into 96-well plates and incubated at 37 °C in a 5% CO_2_ atmosphere. Between day 12 and 14, supernatants were screened by ELISA for binding to *h*Nodal(44–67) peptide and its mutated variant *h*Nodal(44–67)E49A–E50A, in which the glutamic acids 49 and 50 were substituted with two alanine residues. A detailed description of the ELISA procedure is given in the on-line Supplementary Material.

The hybridoma clone named 3D1, with strong reactivity with *h*Nodal(44–67) peptide (but not with the mutated variant) was re-cloned twice by limited dilution and its reactivity was re-confirmed by ELISA. Sub-cloned hybridoma cells were cultured in RPMI-HAT containing 10% FBS and slowly adopted to serum-free medium. The OPTI-MEM medium containing 10% FBS. Adapted cells were cultured in Optimem medium and then transferred to the Bioreactor (Bio Cell Line, Becton Dickinson, Franklin Lakes, NJ, USA) for large scale production.

Isotype of the anti-Nodal antibodies produced by the selected clones was determined by using the commercial kit specific for murine antibodies, in accordance to the manufacturer’s instructions (Pierce Rapid Mouse antibody Isotyping kit, Thermo Scientific, Waltham, MA, USA).

### 3.4. ELISA: Antibody Titration in Mouse Sera

To determine the antibody titer in mouse sera, 96-well ELISA plates were coated with 50 µL of the BSA-conjugated *h*Nodal(44–67), 1 μg/mL in PBS, overnight at 4 °C. The plate was then washed three times with PBS containing 0.05% Tween 20 (PBS-T). Non-specific sites of the plate were blocked with 5% BSA and incubated at R.T. for 120 min. Wells were then washed three times with PBS-T and serum was (50 μL) added to the wells in three-fold serial dilutions starting from 1:100 to 1:100,000. The plate was incubated at 37 °C for 1 h and washed again with PBS-T. Then, 50 μL of a 1:1000 dilution of HRP-conjugated rabbit anti-mouse Ig (1.0 mg/mL, Biorad, Milano, Italy) were added to the wells and incubation was carried out for 1 h at 37 °C. After washing, 100 μL of 2,2-Azino-bis(3-ethylbenzothiazoline-6-sulfonic acid) (ABTS) substrate solution was added to each well. After 15 min, the reaction was stopped by adding 50 μL of 1% SDS solution in water to each well. The Optical Density (OD) was measured at 415 nm by a microplate reader (BioTek, Winooski, VT, USA). Antibody titers were evaluated sufficiently high when the average absorbance values from triplicate wells incubated with immune sera dilutions at 1:10,000 were at least thrice those determined on wells incubated with the pre-immune serum.

### 3.5. ELISA: Screening of Hybridoma Supernatants

For hybridoma supernatants screening, *h*Nodal(44–67) and *h*Nodal(44–67)E49A–E50A were coated at 330 nM diluted in PBS on polystyrene conical flat bottom 96-well plates (Nunc MaxiSorp™, Roskilde, Denmark) at 4 °C overnight (100 µL/well). After incubation, the coated wells were washed three times with PBS containing 0.005% Tween-20 (PBS-T) and non-specific binding sites were blocked by incubating with 1% BSA in PBS (300 µL/well) for 1 h at 37 °C. After washing three times with PBS-T, supernatants of hybridomas diluted in PBS at 33 nM were incubated for 1 h at 37 °C (100 µL/well). After incubation, plates were triple washed with PBS-T and goat anti-mouse HRP-conjugated antibody (1 mg/mL, Blotting Grade Affinity Purified Goat Anti-Mouse IgG (H + L)) diluted 1:1000 in PBS was added as secondary antibody (100 µL/well) and incubated for 1 h at 37 °C. After incubation and three washes with PBS-T, bound antibodies were detected by adding 100 µL/well of freshly prepared *O*-phenylenediamine (0.4 mg/mL) containing H_2_O_2_ (0.4 mg/mL) in 0.1 M citrate buffer (pH 5.2) (SIGMAFAST™ OPD tablet Sigma–Aldrich). The peroxidase reaction was stopped after 5 min with 50 µL/well of 2.5 M H_2_SO_4_ and the optical density was measured at 490 nm, using a microplate reader. The dose-dependent binding of 3D1 to immobilized *h*Nodal(44–67) and *h*Nodal(44–67)E49A–E50A was performed by coating the peptides at 60 nM and using 3D1 at concentrations between 0.1 and 66 nM.

### 3.6. ELISA: Epitope Mapping and Specificity Assay

Peptides used for the epitope mapping and for the specificity assay of the 3D1 mAb were coated at 1.0 µg/mL diluted in PBS; 3D1 mAb, dissolved in PBS, was used as primary antibody at 1.0 µg/mL. The ELISA assays were carried out following the already described procedure.

### 3.7. Purification of Monoclonal Antibodies and F(ab′)_2_/Fab′ Fragments

Antibodies and F(ab′)_2_/Fab′ fragments were purified by standard procedures. After centrifugation at 4000 rpm for 20 min at 4 °C, hybridoma cell-culture supernatants, containing monoclonal IgGs, were filtered on a 0.22 μm filter and loaded onto a HiTrap™ Protein G HP column (GE Healthcare). Purifications were performed on an ÄKTA FPLC™ instrument (GE Healthcare) at a flow rate of 0.6 mL/min, monitoring the absorbance at 280 nm. After washing away the unbound material using PBS (pH 7.4) as loading buffer, bound antibodies were recovered by changing drastically the pH conditions using 100 mM Glycine pH 2.7 as elution buffer. Eluted antibodies were quickly neutralized by adding 1/10 volume 2 M TRIS pH 9.0. To ensure the stability of purified mAbs and of related fragments they were buffer-exchanged into PBS (pH 7.4) and concentrated using an appropriate centrifugal filter (Millipore, Darmstadt, Germany). The concentration was estimated by using the Bradford assay [41]. Purity of concentrated proteins were evaluated by SDS-PAGE and Coomassie blue staining.

Size-exclusion chromatography purifications were performed on a Superdex™ 200 HR 10/300 (GE Healthcare, Piscataway, NJ, USA) gel filtration column, using PBS pH 7.4 as running buffer, at a constant flow rate of 0.5 mL/min, with an elution volume of 25 mL and monitoring the absorbance at 280 nm.

### 3.8. SPR Analyses

All SPR analyses were performed on a Biacore 3000 instrument from GE Healthcare, using CM5 sensor chips and certified HBS buffer (20 mM HEPES, 0.15 M NaCl, pH 7.4, P20, 0.005%). Protein immobilization was carried out following the canonical amine coupling chemistry using the surface immobilization wizard procedure, operating at 5 µL/min [42]. Channels were activated with EDC/NHS for 7 min; for the binding assays of purified mAbs to Nodal protein, *rh*Nodal, opportunely diluted in the pre-selected sodium acetate buffer pH 4.5, was coupled until a 4000 RU level was achieved. Residual reactive groups on the sensor chip surface were deactivated by addition of 1.0 M ethanolamine hydrochloride, pH 8.5. Antibody binding was tested at 20 µL/min injecting solutions of 3D1 (60 µL) in HBS-EP at increasing concentrations (6–100 nM). A 10 mM NaOH solution was used to regenerate the chip surface.

After the single-dose screening, dose-dependent binding analyses were carried out with the two antibodies, 3D1 and 5F10, able to bind the full-length protein. Binding of 3D1 was carried out at concentrations ranging between 6 and 100 nM. For 5F10 higher concentrations were required (100, 250, 500 and 750 nM). Dissociations were monitored for at least 500 s. To carry out the mapping of the 3D1 epitope, the whole antibody was immobilized at 5 µg/mL in 10 mM NaAc buffer pH 5 at a flow rate of 5 µL/min by standard amine coupling chemistry on a CM5 sensor chip. The immobilization level reached with the mAb was about 5350 RU. On another channel of the same sensor chip, the 3D1 Fab′ was immobilized at 10 µg/mL in 10 mM NaAc buffer pH 4.5 at a flow rate of 5 µL/min, following the standard amine coupling chemistry. The amount of immobilized Fab′ was about 2570 RU. Residual reactive groups were deactivated by 1M ethanolamine hydrochloride, pH 8.5. The blank channel was prepared as already described. Real-time binding analyses were performed at a flow rate of 20 µL/min, using HBS-EP buffer, injecting a constant sample volume of 60 µL. Peptides opportunely diluted in HBS-EP buffer were injected at the following concentrations: 0.5, 1, 2.5, 5, 10, and 20 µM. Dissociations were monitored for at least 500 s; the regeneration solution was 5 mM NaOH.

For every single analysis, experimental sensorgrams were aligned, subtracted of blank signals and overlapped. All mathematical manipulations and fitting were performed using the BiaEvaluation software, vers. 4.1 from GE Healthcare. All experimental data gave optimal fittings when processed assuming a 1:1 Langmuir binding interaction.

### 3.9. Antibody Deglycosylation and Pepsinolysis

To improve the splitting of the antibody by pepsin to get functional fragments, 3D1 was first subjected to a deglycosylation reaction with the Peptide-*N*-glycosidase F (PNGase F), supplied by New England Biolabs (Beverly, MA, USA). Following a first preliminary small-scale attempt, the large-scale deglycosylation reaction was performed incubating 1.5 mg of the mAb, diluted in PBS (pH 7.4), with 11 units of PNGase F at 37 °C. The time for optimal deglycosylation was identified as being 48 h.

Pepsinolysis reaction, monitored by SDS-PAGE, was optimized in 20 mM sodium acetate buffer, pH 4.0, using a final *w*/*w* ratio of pepsin (Sigma–Aldrich, Milano, Italy) to antibody 1:25 and incubating the mixture in a 37 °C water bath for 4 h.

### 3.10. Preparation of Fab′ Fragments

Fab′ fragments were produced by reducing selectively the hinge-region disulfide bonds of F(ab′)_2_ using 5 mM 2-Mercaptoethylamine (Thermo Scientific Pierce, Milano, Italy). Twenty mM sodium acetate buffer pH 4.0 was added to the F(ab′)_2_ fragments in PBS pH 7.4 to adjust the pH at 6.0 and 2 mM EDTA was also added. The mixture was incubated for 3 h at 37 °C. After incubation, PBS was added to the mixture to adjust the pH to neutrality. Reduction of F(ab′)_2_ to Fab′ fragments was checked and confirmed by 12% SDS-PAGE gel under non-reducing conditions. After reduction, Fab′ fragments were incubated with 25 mM IAM (Iodoacetamide) for 30 min at room temperature in the dark to block reactive thiols.

### 3.11. LC–ESI-TOFMS Analysis of 3D1 Fab′

The sample analyzed by mass spectrometry was reduced using 20 mM DTT for 1 h at 37 °C. Mass spectrometry analyses were performed on an Agilent 1290 Infinity LC System coupled to an Agilent 6230 time-of-flight (TOF) LC/MS System. The liquid chromatography Agilent 1290LC module was coupled with a photodiode array (PDA) detector and a 6230 time-of-flight MS detector, along with a binary solvent pump degasser, column heater, and autosampler. Based on manufacturer recommendations, the pump was connected to a gradient binary solvent system: solvent A, 0.01% TFA in H_2_O (*v*/*v*) and solvent B, 0.01% TFA in CH_3_CN (*v*/*v*). Chromatographic analyses were performed using a Phenomenex AERIS WIDEPORE reverse phase C4 3.6 mm (50 × 2.1 mm) applying a linear gradient from 25% to 65% solvent B in 15 min. The column flow rate was kept at 0.2 mL/min with the heater at a constant 20 °C. UV spectra were monitored in the range 200–400 nm. Injection volume was 1 μL followed by needle wash. The mass analyzer Agilent 6230 TOF MS was set to operate in positive ion scan mode with mass scanning from 100 to 3200 *m*/*z*. The ion source was upgraded from the original Agilent Jet Stream (AJS) source to the dual-sprayer version for improved reference mass delivery. Nitrogen was used as the drying and nebulizer gas. The instrument acquired data using the following parameters: drying gas temperature, 325 °C; drying gas flow, 10 L/min; nebulizer, 20 psi; sheath gas temperature, 400 °C; sheath gas flow, 11 L/min; VCap. 3.500 V; nozzle, 0 V; fragmentor, 200 V; skimmer, 65 V and octapole RF Vpp was 750. The instrument state was set to extended dynamic range mode (2 GHz). Tuning and calibration were performed before sample runs. Data collection and integration were performed using MassHunter workstation software (version B.05.00). Data were stored in both centroid and profile formats during acquisition. A constant flow of Agilent TOF reference solution through the reference nebulizer allowed the system to continuously correct for any mass drift by using two independent reference lock-mass ions, purine (*m*/*z* 119.03632) and HP-922 (*m*/*z* 922.000725), to ensure mass accuracy and reproducibility. Target compounds were detected and reported from accurate-mass scan data using Agilent MassHunter Qualitative software.

### 3.12. Competition between 3D1 Antigen Peptide and Endogenous Nodal Protein

Thirty micrograms of H9 and H14 human embryonic stem cell total protein lysates, obtained as previously described [17,43], were separated on a 15% SDS-PAGE gel under reducing conditions and the resolved proteins were transferred onto a PVDF membrane. Fifty ng of *rh*Nodal protein were used as positive control. The western blotting procedure was carried out as previously described. Membranes were blocked, then probed with the 3D1 alone at 4.0 µg/mL in 2.5% NFDM in TBS-T and with the 3D1 mAb at 4.0 µg/mL pre-incubated with its antigen, the *h*Nodal(44–56) peptide at 10 µg/mL. Detection was achieved with GAM-HRP (Bio-Rad) 1:1000 (1.0 µg/mL). Blots were developed with Enhanced Chemiluminescence Western Blot Substrate (Pierce, Rockford, IL, USA) following the manufacturer’s instructions and were acquired by using a Chemidoc XRS video densitometer (Bio-Rad, Hercules, CA, USA).

### 3.13. Detection of Endogenous Human Nodal in Melanoma Cells

Western blot analyses for the detection of endogenous Nodal protein were performed using the following human skin malignant melanoma cell lines: PES, A-375, SK-MEL, WM 266 and LCM. HEK-293 (human embryonic kidney) cells and 100 ng of *rh*Nodal protein were used as positive controls. Cells were lysed performing three freeze/thaw cycles in 50 mM Tris–HCl pH 7.5, 500 mM NaCl, 1% NP-40, 10 mM EDTA, 1 mM MgCl_2_, 1 mM CaCl_2_, 10% Glycerol, with an added cocktail of protease inhibitors (Roche, city, Switzerland) and PMSF (phenyl-methanesulfonylfluoride). Cell lysates were cleared by centrifugation at 12,000 rpm for 20 min at 4 °C and the supernatants were quantified by Bradford assay. Fifty µg of cell lysate from each sample were applied to a 15% SDS-PAGE gel under reducing conditions and electrophoresed for 3 h at 80 V. Separated proteins were transferred onto a polyvinylidene difluoride (PVDF) membrane (Millipore), previously soaked in methanol 100% for 5 min. The blot was blocked with 5% milk (NFDM) in Tris-buffered saline containing 0.1% Tween-20 (TBS-T) for 1 h at room temperature under shaking; next, the membrane was probed overnight at 4 °C with 3D1 mAb as primary antibody at 2 µg/mL in 2.5% NFDM in TBS-T under shaking. The membrane was then triple-washed with TBS-T and incubated with GAM-HRP as secondary antibody diluted 1:1000 in TBS-T for 1 h at room temperature under agitation. The target protein was detected using the enhanced chemi-luminescent substrate method with the SuperSignal West Pico kit provided by Pierce Chemical Co. (Rockford, IL, USA), following the manufacturer’s instructions. Signals were acquired with the ChemiDoc™ XRS video densitometer (Bio-Rad, Hercules, CA, USA), using the Quantity One^®^ software (Bio-Rad, Milano, Italy).

### 3.14. FACS Analyses

LCP, A375 and WM266 melanoma cells were collected by centrifugation. After aspiration of supernatant, the cells were resuspended in 500 µL of PBS and formaldehyde was added to obtain a final concentration of 4%. Cells were fixed for 10 min at room temperature. After fixation, cells were permeabilized by adding 500 µL of PBS-Tween 20.

1 × 10^6^ cells were aliquoted into each vial; 1 mL of PBS (incubation buffer) was added to each vial and cells were washed by centrifugation at 4000 rpm for 5 min at 4 °C. Cells were resuspended in 100 µL of solution of 3D1 anti-Nodal antibody (primary antibody), diluted in PBS at the following concentrations: 0.1, 0.5, 1.0 and 2.0 µg/mL. A standard IgG_1_ isotype was used as negative control. Cells were incubated with the primary antibody for 1 h at 4 °C under shaking. After incubation, cells were washed by centrifugation in 1 mL of PBS, resuspended and incubated with a FITC-conjugated anti-mouse antibody (secondary antibody), diluted 1:1000 (1.0 µg/mL) in PBS, for 40 min at 4 °C in the dark under stirring. Cells were washed by centrifugation with 1 mL of incubation buffer, re-suspended in 500 µL of PBS and analyzed on flow cytometer. Fluorescence was evaluated using the BD FACScalibur™ System.

## 4. Conclusions

Nodal, a member of the TGF-β superfamily, is a potent morphogen and regulator of cell fate in both embryological and adult systems.

Its expression is largely restricted to very early progenitor and reproductive cell types, whereas it is almost absent in adult tissues and normal cells, including melanocytes. Its re-expression in the adults is strongly associated with tumorigenesis, indeed Nodal levels are much higher in tumor cells and tissues where it supports uncontrolled cell growth, proliferation and differentiation [8].

Several studies have reported that Nodal expression positively correlates with melanoma tumor progression toward a metastatic phenotype; moreover, its over-expression is paralleled by that of the co-receptor Cripto-1, suggesting the occurrence of tumor growth mechanisms supported by the concomitant presence of both proteins and by their mutual interaction with the activin-like receptor complex [5,9,10,11,12,13,22,23].

In light of this evidence, Nodal is considered a promising diagnostic and prognostic marker and a very important and interesting new therapeutic target for several types of cancers [4]. So far, exogenous Nodal inhibitors contrasting its anti-apoptotic and tumorigenic activity have not been reported. Nodal activity down-regulation, which dampens both Cripto-1-dependent and independent signalling, has been only achieved by using antisense technology, an approach that has largely contributed to elucidate melanoma-related functions [9].

To develop pharmaceutical inhibitors and reagents useful for its detection in tumor tissues, we have targeted Nodal with monoclonal antibodies *ad hoc* generated to bind the Cripto-1 interaction surface. mAbs have been developed against a Nodal peptide encompassing residues 44–67 [21,31].

We have privileged the approach based on mAbs because they are readily accessible by the hybridoma technology, which generally affords antibodies in a straightforward manner and in high amounts. Antibodies from supernatants of stabilized hybridoma cells are also easily purified and characterized by current chromatographic techniques and, most importantly, are in most cases endowed with a sufficiently high affinity and specificity for the target antigens.

The anti-Nodal mAbs have been generated and screened following a novel, subtractive approach to select only those able to bind the wild type peptide antigen and not a variant mutated containing two mutated alanine on E49 and E50, two specific residues directly involved in the binding with Cripto-1 [21,31]. mAbs selected by this approach had the potential to interact with high specificity with these and other surrounding residues thereby working as both Nodal binders and inhibitors of the interaction with Cripto-1. Antibody 3D1 also recognized with high affinity the full-length native protein. The ability of 3D1 to recognize native and non-native Nodal under different experimental conditions has been here proven by a variety of techniques, therefore the antibody can be seen as a new analytical tool for detecting the protein in tissues and in biological fluids where it is expressed and in theragnostic programs aimed at developing Nodal inhibitors. Remarkably, 3D1 also blocks *in vitro* the interaction of Nodal with Cripto-1, a feature of particular relevance in the context of tumor development mechanisms involving the activation of the Smad2/3 axis [32]. As recently shown [20,21], Nodal also binds to ALK4 and the region of binding seems to be shared with Cripto-1 and ALK7, two well-established Nodal (co)-receptors. On this basis we could speculate that 3D1 has the potential to also inhibit the interaction of Nodal with other receptors thus strengthening inhibition of Smad 2/3 activation and potentiating the anti-tumoral activity of the antibody [32].

Collectively analytical and functional data show that the anti-Nodal 3D1 mAb is a promising reagent for the detection of Nodal under different conditions and in different tissues. The Smad 2/3 and MAPK neutralizing activity also suggests it is a promising novel biotherapeutic agent.

The generation of smaller functional fragments (Fab, ScFv), together with a step of affinity improvement and humanization, will provide a unique and invaluable reagent for the treatment, alone or in combination with other drugs, of cutaneous melanoma and other relevant cancer diseases.

## Supplementary Materials

Supplementary materials can be found at http://www.mdpi.com/1422-0067/16/09/21342/s1.
