# Supplementary material for: New Anti-Nodal Monoclonal Antibodies Targeting the Nodal Pre-Helix Loop Involved in Cripto-1 Binding
Source: Int J Mol Sci. 2015 Sep 7;16(9):21342–62. doi: 10.3390/ijms160921342 (PMC4613256; doi:10.3390/ijms160921342)
Supplement: Supplementary file 1 [file ijms-16-21342-s001.pdf]

Supplementary Information

Table S1. Anti-Nodal mAbs screened and K_D values determined for the binding to *rh*Nodal functionalized sensor chip. NB: No Binding.

**mAbs**	**Binding to *rh*Nodal**
3D1	K_D = 1.4 nM
5F10	K_D = 84 nM
1B4	NB
9B9	NB
10B12	NB
2D12	NB

Table S2. Association and dissociation rate constants. (**a**) K_D values determined for the binding of the 3D1 mAb to *rh*Nodal functionalized sensor chip; (**b**) K_D values determined for the binding of the 5F10 mAb to *rh*Nodal-functionalized sensor chip; (**c**) K_D values determined for the binding of the 3D1 F(ab′)2 to *rh*Nodal functionalized sensor chip; (**d**) K_D values determined for the binding of the 3D1 Fab′ to *rh*Nodal functionalized sensor chip.

(**a**)

3D1 mAb	k_a (1/Ms)	k_d (1/s)	K_D (M)	SD *
6 nM	1.28×10^{6}	6.29×10^{-4}	4.91×10^{-10}	0.0129
12 nM	9.79×10^{5}	6.83×10^{-4}	6.98×10^{-10}	0.0504
25 nM	6.42×10^{5}	6.22×10^{-4}	9.68×10^{-10}	0.1650
50 nM	3.61×10^{5}	6.97×10^{-4}	1.93×10^{-9}	0.3310
100 nM	2.12×10^{5}	6.44×10^{-4}	3.03×10^{-9}	1.0700
Average	6.95×10^{5}	6.55×10^{-4}	1.42×10^{-9}	0.3260

(**b**)

5F10 mAb	k_a (1/Ms)	k_d (1/s)	K_D (M)	SD *
100 nM	2.60×10^{4}	8.16×10^{-4}	3.14×10^{-8}	0.0246
250 nM	1.81×10^{4}	1.08×10^{-3}	5.97×10^{-8}	0.0818
500 nM	1.31×10^{4}	1.33×0^{-3}	1.02×10^{-7}	0.1480
750 nM	9.40×10^{3}	1.30×10^{-3}	1.38×10^{-7}	0.0286
Average	1.91×10^{4}	1.08×10^{-3}	8.28×10^{-8}	0.0708

(**c**)

F(ab′)2	k_a (1/Ms)	k_d (1/s)	K_D (M)	SD *
25 nM	4.40×10^{5}	1.13×10^{-3}	2.57×10^{-9}	0.0214
50 nM	2.77×10^{5}	1.35×10^{-3}	4.87×10^{-9}	0.0578
100 nM	1.87×10^{5}	1.63×10^{-3}	8.71×10^{-9}	0.323
250 nM	8.21×10^{4}	1.67×10^{-3}	2.03×10^{-8}	0.446
500 nM	4.58×10^{4}	1.84×10^{-3}	4.02×10^{-8}	1.63
Average	2.06×10^{5}	1.52×10^{-3}	1.53×10^{-8}	0.496

(**d**)

Fab′	k_a (1/Ms)	k_d (1/s)	K_D (M)	SD *
**25 nM**	3.42×10^{5}	2.02×10^{-3}	5.91×10^{-9}	0.0347
**50 nM**	1.59×10^{5}	2.62×10^{-3}	1.65×10^{-8}	0.0245
**75 nM**	1.50×10^{5}	1.92×10^{-3}	1.28×10^{-8}	0.0949
**100 nM**	1.18×10^{5}	1.56×10^{-3}	1.32×10^{-8}	0.0909
**200 nM**	7.59×10^{4}	2.31×10^{-3}	3.04×10^{-8}	0.145
**average**	$\mathbf{1.69 \times 10^{5}}$	$\mathbf{2.09 \times 10^{-3}}$	$\mathbf{1.58 \times 10^{-8}}$	**0.0780**

* SD: Standard Deviation.

Table S3. Nomenclature and amino acid sequence of hNodal peptides screened in the epitope mapping study and KDvalues determined for the binding of the positive peptides to 3D1 mAb/Fab′ functionalized sensor chip. No fitting means that fitting of binding association curve did not converge to any value.

***h*Nodal Peptide 0.5–20 µM**	**Sequence**	**K_D *vs.* 3D1 mAb**	**K_D *vs.* 3D1 Fab′**
(44–67)	PNPVGEEFHPTNHAYIQSLLKRYQ	613 nM	590 nM
(44–67)E49A–E50A	PNPVGAAFHPTNHAYIQSLLKRYQ	NO BINDING	NO BINDING
(44–67)P46A–V47A	PNAAGEEFHPTNHAYIQSLLKRYQ	NO BINDING	NO BINDING
(44–56)	PNPVGEEFHPTNH	413 nM	371 nM
(52–60)	HPTNHAYIQ	NO BINDING	NO BINDING
(56–67)	AYIQSLLKRYQ	NO BINDING	NO BINDING

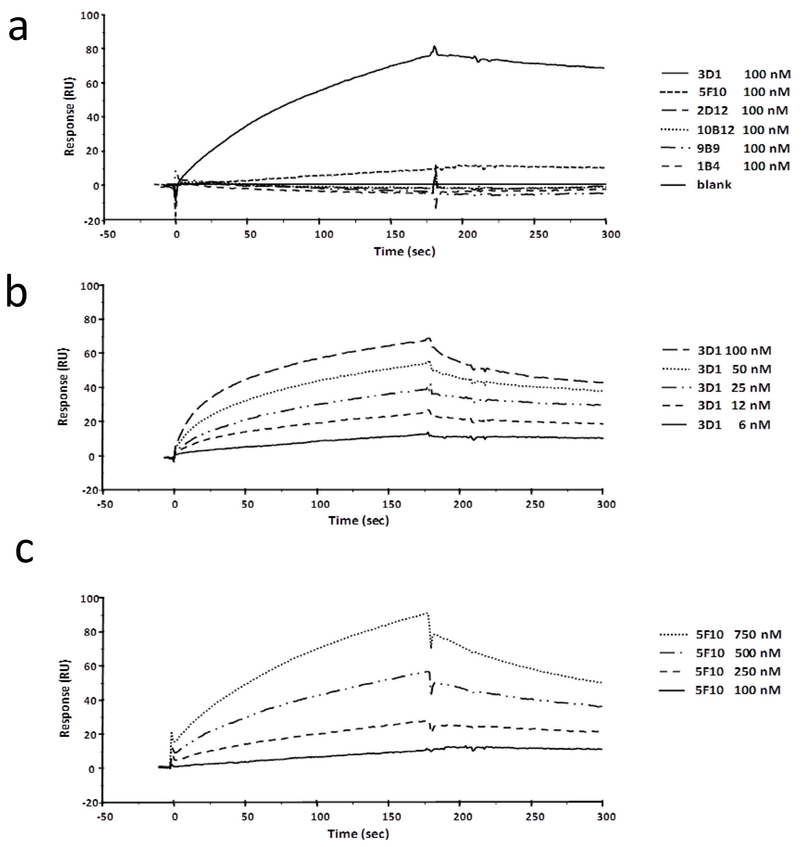

Figure S1. (**a**) Screening of anti-Nodal monoclonal antibodies; Overlay plot of SPR sensorgrams showing the interaction between 3D1 and 5F10 mAbs with rhNodal immobilized on a CM5 sensor chip. The interaction was monitored at concentrations of mAb ranging between 6 and 100 nM for 3D1 (**b**) and 100 and 750 nM for 5F10 (**c**) obtaining dose-dependent binding curves.

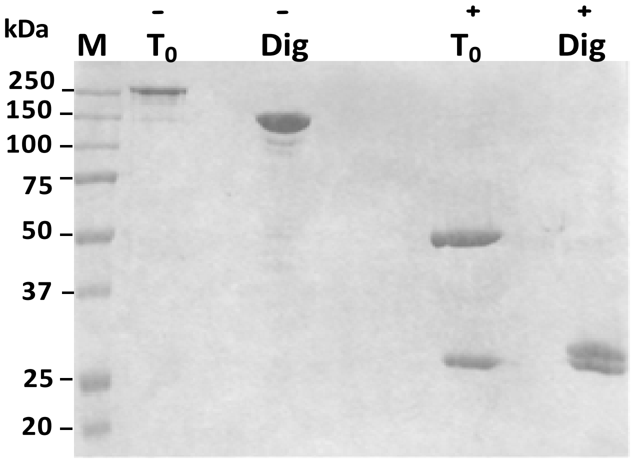

Figure S2. 12% SDS-PAGE analysis under non reducing (−) and reducing (+) conditions of products obtained following digestion of the 3D1 mAb with Pepsin; T_0: 3D1 antibody; Dig: proteolytic digest of 3D1 after 6 h.

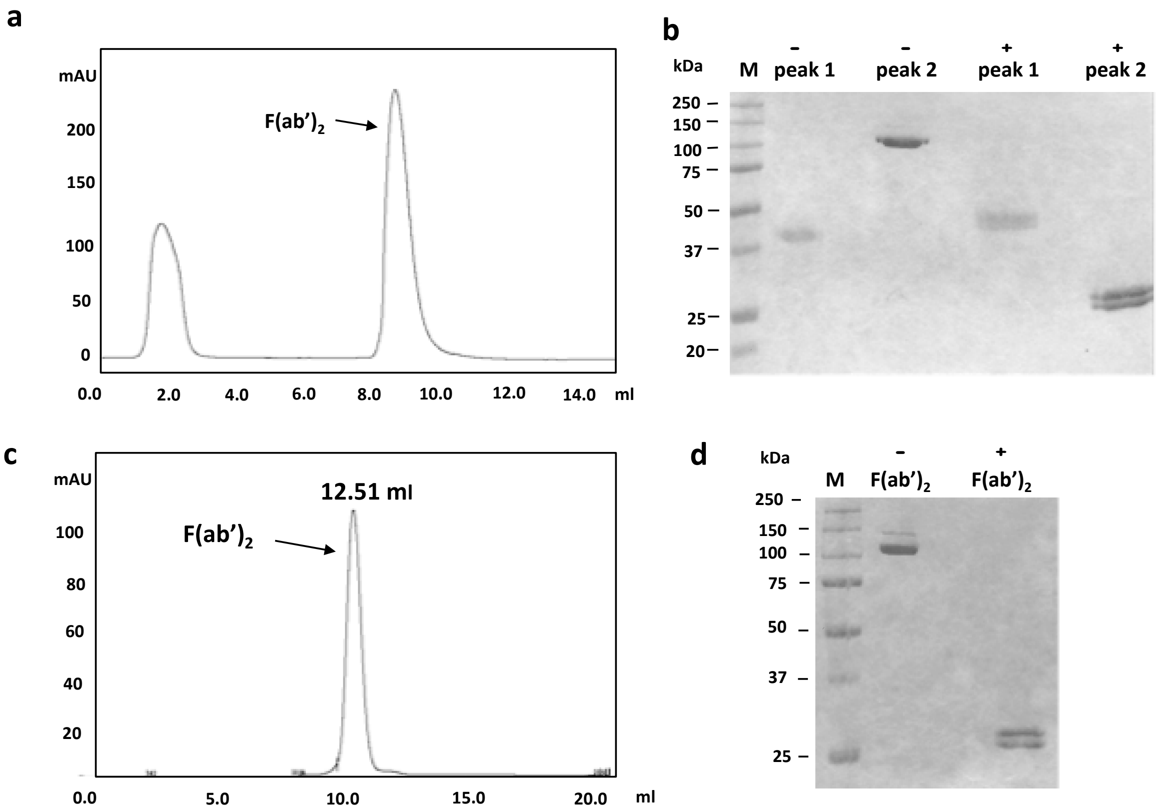

Figure S3. (**a**) Chromatogram of Protein G affinity purification and (**b**) SDS-PAGE analysis of products obtained by pepsin digestion; (**c**) SEC profile with the retention volume and (**d**) SDS-PAGE analysis of F(ab′)2 obtained by pepsin digestion.

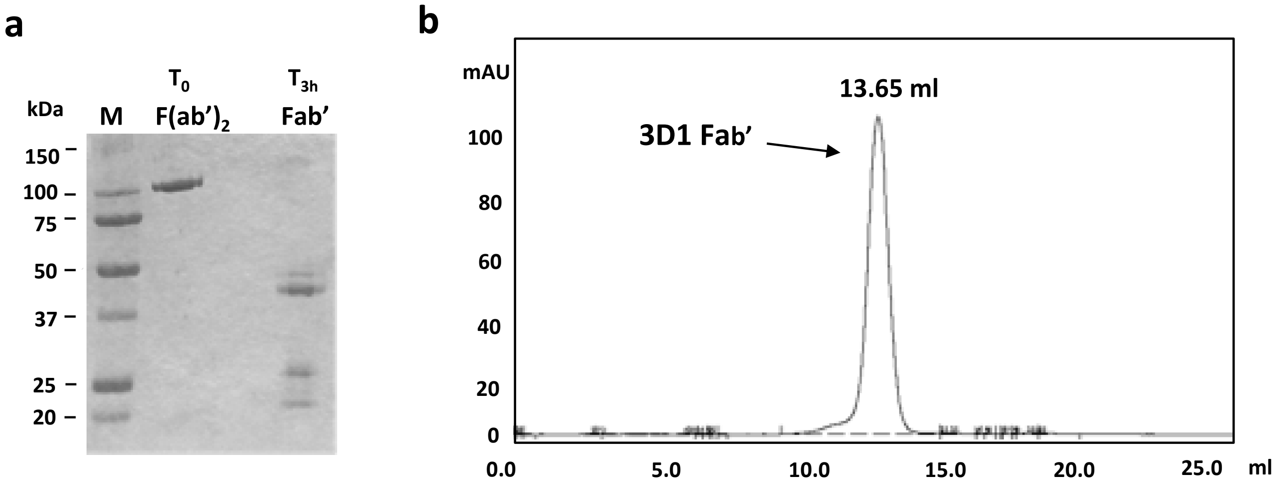

Figure S4. (**a**) 12% SDS-PAGE analysis under non reducing conditions of the F(ab′)2 and F(ab′)2 reduced to Fab′; (**b**) SE-chromatographic profile of the Fab′.

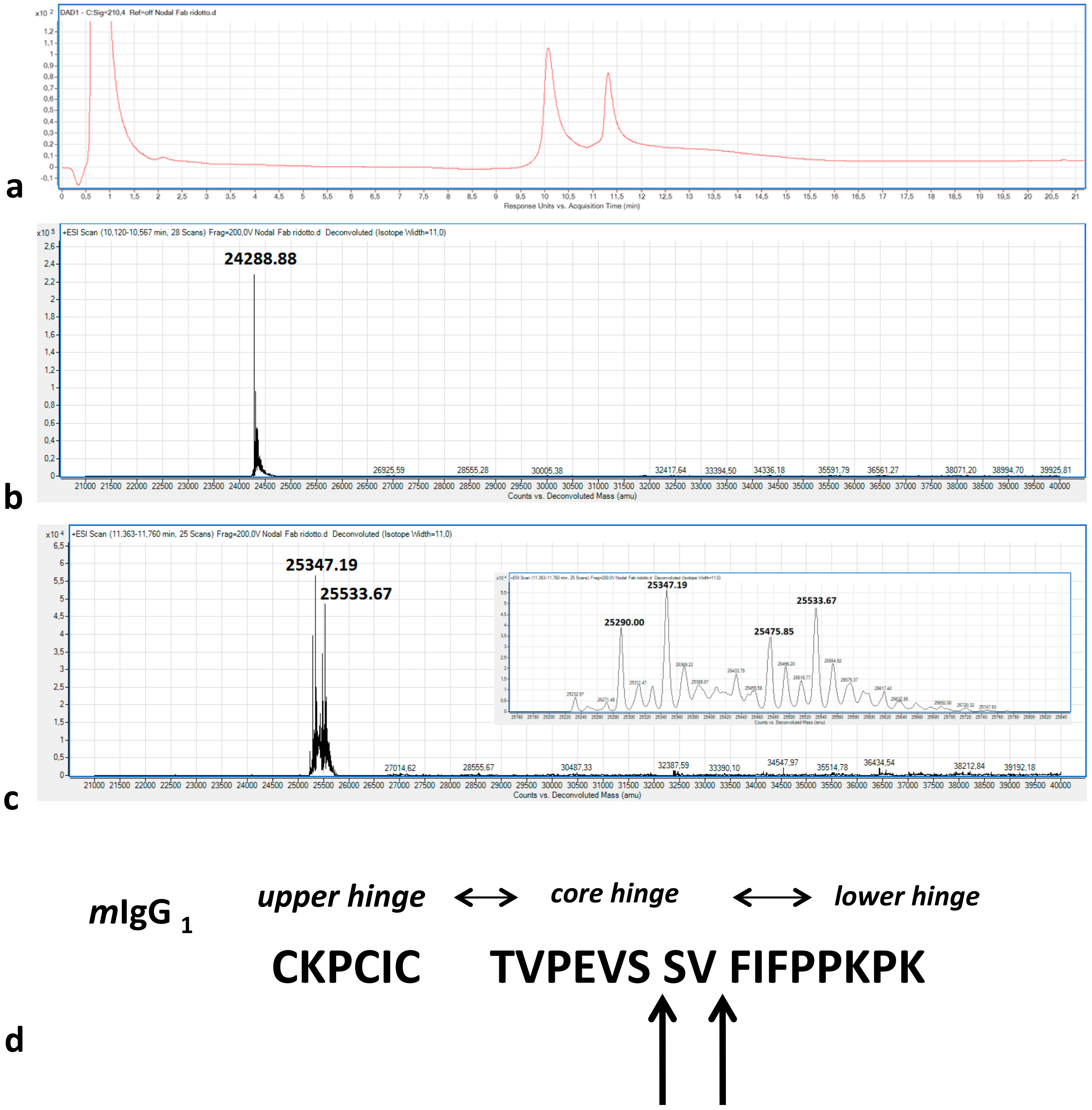

Figure S5. LC–MS analysis of the reduced and alkylated 3D1-Fab′: Chromatographic profile (**a**) of the two separated chains: the Light Chain (LC) eluted at 10.07 min and the 3D1-Fab′ Heavy Chain (HC), eluted at 11.31 min. Deconvolution of mass spectra obtained for both peaks are also reported. LC exhibited a single and homogeneous product (**b**), whereas HC showed multiple products deriving from pepsin unspecific cleavage on the hinge region (**c**). Schematic representation of the supposed cleavage sites on the mouse IgG1 heavy chain (**d**).

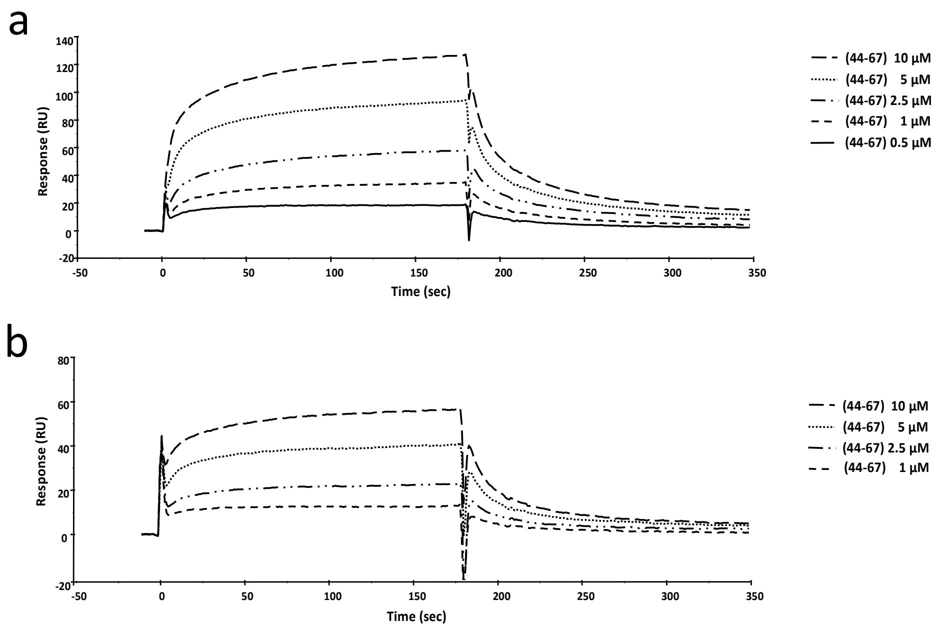

Figure S6. Overlay plot of SPR sensorgrams showing the binding of *h*Nodal(44–67) with both 3D1 mAb (**a**) and its Fab′ fragment (**b**) immobilized on a CM5 sensor chip. The interaction was monitored at concentrations of peptide ranging between 0.5 and 10 µM for the binding of *h*Nodal(44–67) to 3D1 and between 1 and 10 µM for the binding to the Fab′ fragment.

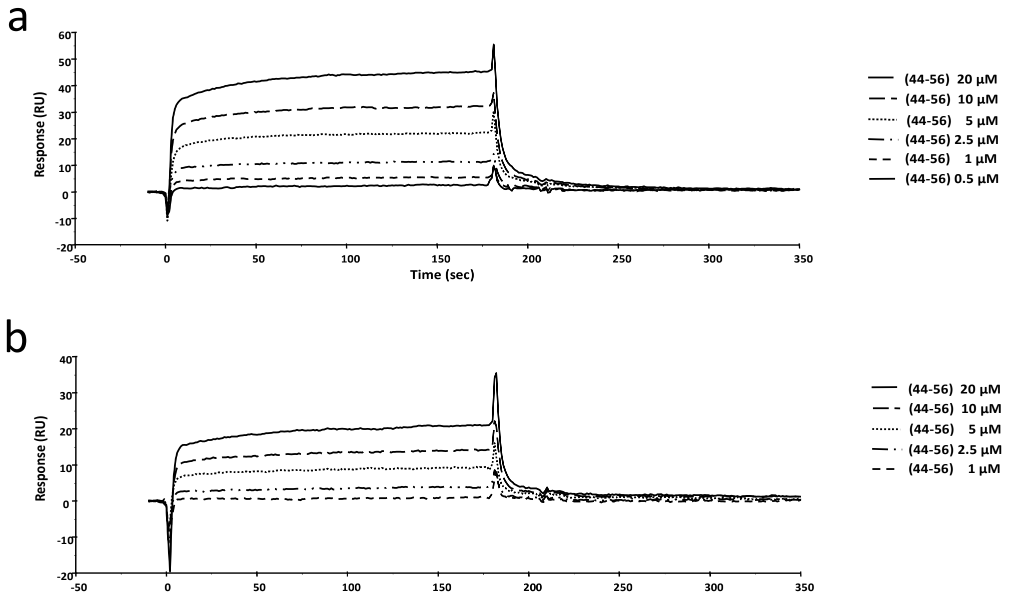

Figure S7. Overlay plot of SPR sensorgrams showing the binding of *h*Nodal(44–56) with both the 3D1 mAb (**a**) and its Fab′ fragment (**b**) immobilized on a CM5 sensor chip. The interaction was monitored at concentrations of peptide ranging between 0.5 and 20 µM for 3D1 and between 1 and 20 µM for the Fab′ fragment, obtaining dose-dependent binding curves.

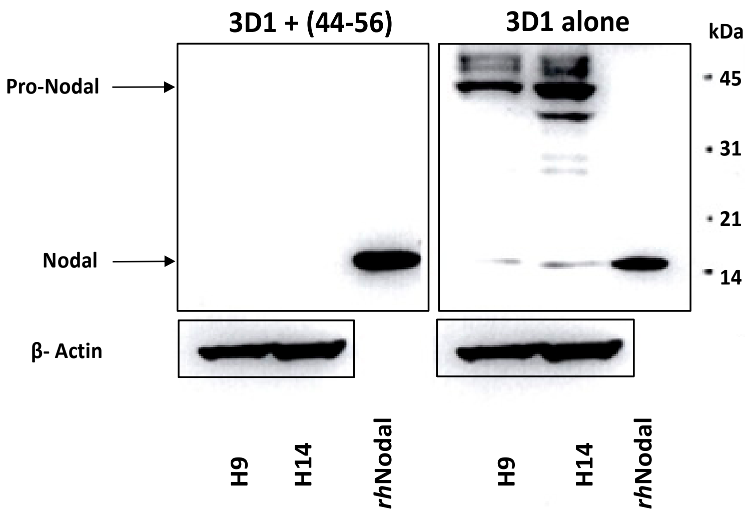

Figure S8. Competition assay between endogenous Nodal in human embryonic stem cells lysates and the Nodal peptide corresponding to the 3D1 epitope. 3D1 was used at 4 µg/mL and *h*Nodal(44–56) at 10 µg/mL.
